# A technical evaluation of the Instrumentation Laboratory Multistat I Analyser

**DOI:** 10.1155/S1463924681000059

**Published:** 1981

**Authors:** J. F. Stevens, D. J. Allan, D. A. Rickwood, B. P. Ager

**Affiliations:** 1 Courtauld Institute of Biochemistry The Middlesex Hospital Medical School Riding House Street London W1 UK; 2 The Department of Chemical Pathology Mount Vernon Hospital Northwood Middlesex UK; 3 The Department of Health and Social Security Scientific and Technical Branch 14 Russell Square London WCIB 5EP UK


### A technical evaluation of the Instrumentation Laboratory Multistat I Analyser

**J.F. Stevens**
*Courtauld Institute of Biochemistry, The Middlesex Hospital Medical School, Riding House Street, London W1*

**D.J. Allan, D.A. Rickwood**
*The Department of Chemical Pathology, Mount Vernon Hospital, Northwood, Middlesex*

**B.P. Ager***
*The Department of Health and Social Security, Scientific and Technical Branch, 14 Russell Square, London WC1B 5EP. UK.*

### Introduction

In most clinical chemistry laboratories the workload involved in the measurements of electrolytes is the major repetitive item. The average throughput for most laboratories ranges from one hundred to one hundred and fifty profiles per day. The Instrumentation Laboratory** (IL) Multistat I system has been designed to cope with this level of work.

The handling and reporting of over one thousand test results a day poses many problems of data handling, reporting and quality control procedures. In order to cope with this, the Multistat I incorporates a hardwired computer which, not only controls all operations of the analyser, but also accepts input of patient data and produces individual patient reports, cumulative reports and details of quality control performance.

Analytical channels are provided for sodium, potassium, chloride, total $CO_2$, urea, creatinine and glucose, lithium may also be run separately. Both plasma and urine samples may be analysed.

This report describes the technical performance of the system located at Mount Vernon Hospital, Northwood during the first three months after its installation in November 1978.

### Materials and Methods

### The Multistat I

The instrument has been the subject of a detailed description by Gibson et al [1].

The analytical system comprises three analytical modules (IL743, IL446 and IL919) and an autosampler (IL461). The modules were designed for "stat" or automatic operation and are now available as a multichannel analyser with computer control and data handling.

### IL743 Autocal flame photometer

This instrument embodies the features of previous IL flame photometers with the addition of push-button automatic calibration and a noise detector which only allows readings to be made when the signal is stable.

### IL446 chloride total $CO_2$ analyser

In this instrument chloride is measured by a colorimetric method using a ferric nitrate/mercuric thiocyanate reagent. Sample and reagent are aspirated by a peristaltic pump, mixed and passed through a small flow cell with a fibre optic light source, interference filter and solid state detector. Total $CO_2$ is measured after automatic dilution and acidification using a $PCO_2$ electrode. The carbon dioxide released is measured directly.

### IL919 glucose/urea/creatinine analyser

This is a discretionary, three channel kinetic analyser using glucose oxidase/peroxidase to measure glucose, ureaseglutamate dehydrogenase to measure urea and alkaline picrate to measure creatinine.

Sample volumes are measured by slide valves. Sample is mixed with reagents and the reaction measured kinetically for 16 seconds in each of three cuvettes. Mixing of reagent and sample is achieved by rotation of cuvettes followed by centrifugal removal of the contents for cleaning purposes.

### IL461 Autosampler

The sampler uses circular plates holding up to 50 cups. It automatically detects the plate number and sample position and identifies calibration standards. The sampler has separate probes for each of the analytical modules described above. The 919 sample line is flushed and dried between samples.

The sampling rate depends on the channels in use with a maximum of 60 per hour for all seven channels.

### Computer hardware

The operations of the modules are controlled by a Computer Automation LSI - 3/05 CPU. Operators can communicate with the system using the visual display unit (VDU) and keyboard. The programs and analytical data are stored on one of the two flexible diskettes provided with instrument.

A fast serial printer (200 cps) is provided for output and produces worksheets, print backup of analytical results, patient reports and quality control statistics.

The analytical modules and computer are located in an instrument console which also houses a sample loader. The loader allows correct loading of the sample which is being entered at the keyboard.

### Operation

In order to operate the system it is necessary to prepare the appropriate reagents and to prime each module manually. The working program is loaded from the diskette, date and time are entered and a plate format program is run to load the plate with standards and patient samples. Patient identification details are also entered at this stage. Quality control samples are included on each plate and drift controls may be sampled in any position as decided by the operator. As the analyses proceed, a print backup is generated. The sampler will stop and the operator alerted if an unsaitable calibration is obtained or if the drift standards are outside the range expected.

**To whom correspondence should be addressed*

***Instrumentation Laboratory (UK) Ltd, Kelvin Close, Birchwood Science Park, Warrington, Cheshire.*

Quality control data can be examined at any stage on the VDU. All analytical data is stored on the appropriate diskette. Final reports can be produced on pre-printed stationery if required. Additionally, calculated osmolality and anion gap may be reported.

### Clinical laboratory evaluation

The evaluation protocol used was based on that described by Broughton et al (2).

*Precision* was measured at three levels of analyte over a period of twenty consecutive working days using the sera available for Technicon† SMA post maintenance performance assessment. Two trays of randomly placed high medium and low sera were analysed with minimum interruption each day.

*"Accuracy"* was assessed in two ways; by comparison with stated values for commercial quality control material and comparison with results on patient samples analysed by both a Vickers†† M300 analyser and a Technicon SMA.

*Linearity* was measured by analysing a series of dilutions of a concentrated stock of aqueous of serum pools.

*Carryover* was assessed by analysing sets of successive high and low triplicates over a period of several days.

During the period of the evaluation a note was made of the instrument reliability, usefulness of the manufacturer's training and instruction manual and safety aspects.

### Results

An initial performance assessment of the system was carried out. A plate of pooled sera was analysed without drift control followed by a second plate of the same material with drift controls at ten sample intervals. The results are shown in Tables 1 and 2.

Without drift control, all channels except chloride, total $CO_2$ and "anion gap" shown satisfactory precision. When drift control was applied, it can be seen that there was little difference in the accuracy or precision of glucose, urea or creatinine indicating that control is not required. The other channels have been drift corrected and it can be seen that the precision of sodium and potassium was not as good as without drift control but the mean results were closer to the correct values this illustrates the problem of over compensation by drift control. The imprecision was thought to be due to carry-over so it was decided to place an extra drift material preceding the actual drift control. The chloride and total $CO_2$ gave

*†Technicon Instruments Co Ltd, Evans House, Hamilton Close, Basingstoke, Hants, RG21 2YE, UK.*

*†† Vickers Ltd, Medical Engineering, Priestly Road, Basingstoke, Hampshire RG24 9NP, UK.*

**Table 1. Initial performance of the system on replicate pooled sera without drift control.**

| Channel | | n | Mean | SD | CV% |
|---|---|---|---|---|---|
| Sodium | mmol/l | 40 | 148.1 | 1.39 | 0.93 |
| Potassium | mmol/l | 40 | 5.40 | 0.05 | 0.92 |
| Chloride | mmol/l | 40 | 110.4 | 5.83 | 5.27 |
| Total $CO_2$ | mmol/l | 40 | 25.8 | 1.25 | 4.8 |
| Glucose | mmol/l | 40 | 12.3 | 0.13 | 1.11 |
| Urea | mmol/l | 40 | 18.3 | 0.09 | 0.53 |
| Creatinine | µmol/l | 40 | 426.6 | 3.58 | 0.83 |
| Anion Gap | mmol/l | 40 | 16.9 | 5.4 | 31.0 |
| Osmolality | mosm/kg | 40 | 320.1 | 6.97 | 2.17 |

**Table 2. Initial performance of the system on replicate sera with drift control.**

| Channel | | n | Mean | SD | CV% |
|---|---|---|---|---|---|
| Sodium | mmol/l | 40 | 140.1 | 1.83 | 1.3 |
| Potassium | mmol/l | 40 | 5.18 | 0.12 | 2.3 |
| Chloride | mmol/l | 40 | 100.5 | 2.81 | 2.79 |
| Total $CO_2$ | mmol/l | 40 | 23.9 | 0.25 | 1.08 |
| Glucose | mmol/l | 40 | 12.36 | 0.13 | 1.09 |
| Urea | mmol/l | 40 | 18.29 | 0.14 | 0.75 |
| Creatinine | µmol/l | 40 | 435.6 | 6.3 | 1.45 |
| Gap | mmol/l | 40 | 20.6 | 4.04 | 19.6 |
| Osmolality | mosm/kg | 40 | 300.0 | 3.2 | 1.06 |

**Table 3. Average overall precision as root-mean-square standard deviation and coefficient of variation for each channel over 20 day precision trial. Values are also given for within plate precision over this period.**

| Channel | N | Mean | RMS SD | CV % | RMS SD (Plate) | CV % |
|---|---|---|---|---|---|---|
| Sodium mmol/l | 393 | 149.72 | 1.63 | 1.10 | 1.38 | 0.93 |
| | 398 | 143.09 | 1.58 | 1.11 | 1.41 | 0.99 |
| | 395 | 124.33 | 1.35 | 1.09 | 1.20 | 0.97 |
| Potassium mmol/l | 388 | 7.48 | 0.09 | 1.24 | 0.07 | 1.02 |
| | 393 | 4.63 | 0.05 | 1.25 | 0.05 | 1.14 |
| | 391 | 3.11 | 0.03 | 1.15 | 0.03 | 1.01 |
| Chloride mmol/l | 392 | 122.87 | 3.93 | 3.20 | 3.68 | 3.00 |
| | 392 | 108.40 | 3.53 | 3.26 | 3.46 | 3.19 |
| | 390 | 81.36 | 2.60 | 3.20 | 2.50 | 3.08 |
| Total $CO_2$ mmol/l | 176 | 28.03 | 1.18 | 4.23 | 1.04 | 3.72 |
| | 176 | 24.04 | 1.12 | 4.70 | 0.87 | 3.64 |
| | 178 | 18.17 | 0.97 | 5.38 | 0.74 | 4.08 |
| Glucose mmol/l | 398 | 11.11 | 0.19 | 1.79 | 0.15 | 1.39 |
| | 399 | 5.86 | 0.11 | 2.01 | 0.10 | 1.75 |
| | 397 | 3.28 | 0.11 | 3.40 | 0.10 | 3.17 |
| Urea mmol/l | 218 | 19.32 | 1.00 | 5.18 | 0.46 | 2.38 |
| | 219 | 9.88 | 0.50 | 5.10 | 0.17 | 1.78 |
| | 217 | 4.97 | 0.20 | 5.00 | 0.08 | 1.98 |
| Creatinine µmol/l | 398 | 552.41 | 20.02 | 3.62 | 10.08 | 1.83 |
| | 399 | 313.64 | 11.94 | 3.81 | 6.75 | 2.15 |
| | 397 | 110.17 | 7.17 | 6.52 | 5.95 | 5.41 |
| Anion Gap | 382 | 18.40 | 4.55 | 24.76 | 4.25 | 23.11 |
| | 385 | 25.79 | 4.20 | 16.29 | 4.15 | 16.11 |
| | 385 | 34.63 | 3.22 | 9.32 | 3.15 | 9.12 |
| Osmolality msom/kg | 380 | 332.49 | 4.73 | 1.43 | 4.26 | 1.28 |
| | 384 | 291.11 | 4.24 | 1.46 | 3.92 | 1.35 |
| | 385 | 230.28 | 3.46 | 1.50 | 3.04 | 1.32 |

much better precision after drift control. There was an improvement in the precision of the calculated values but the "anion gap" precision was still not acceptable.

**Precision**

Precision was assessed over the twenty day period using a fresh test kit of high, medium and low sera each day. The results are presented in Table 3. A problem was encountered with the diluent for the sera which was a solution of ammonium carbonate. Since this would adversely affect the urease method, and its absence would considerably reduce the total $CO_2$ value, it was used on alternate days with deionised water being used on the others. The result is a 50% reduction in the number of data points available for urea and total $CO_2$ precision. This is similar to the performance seen on the initial performance assessment. Again the calculated "anion gap" is very imprecise.

**"Accuracy"**

Comparison was made between stated values for eleven commercial quality control sera and the values obtained with the Multistat I. The sera used are shown in Table 4 together with the quoted and assay results. Many manufacturers use ammonium carbonate solution to reconstitute freeze dried sera in order to obtain good results for total $CO_2$. The use of such a diluent on this system would affect the urea result. There is one apparent discrepancy in the compared values. This is in the creatinine value for Versatrol normal.

The sample was analysed many times on the system and consistently obtained a result of 238 μmol/l. Analysing the sample on other systems gives results close to the quoted value (133 μmol/l). This apparent error cannot be explained in analytical terms. Obviously there is an interfering substance present in the material which only affects the kinetic assay.

Comparisons were also made for urine sodium, potassium and creatinine using commercially available urine controls. The results are shown in Table 5.

A second aspect to the "accuracy" study was to compare the results obtained on patient samples analysed by the Multistat I and by other analysers. Two studies were carried out. Samples which had been analysed on a Vickers M300 were collected from the Middlesex Hospital and analysed on the Multistat I. Samples which had been analysed on a Technicon SMA Plus at St Albans City Hospital were similarly analysed. The results are shown in Tables 6 and 7. The different intercepts from day to day on the chloride channel (+37 to -2.9) were later traced to the reagent difficulties which were cured by de-gassing the reagent before use. Due to the problems of transport for samples with bicarbonate values it was decided to perform this comparison locally with the method used in the laboratory on a continuous flow system. The results are shown in Table 8. Calculated osmolality was compared with true osmolality as measured on a vapour phase Wescor Osmometer. The results are shown in Table 8. Lithium measurements are not normally made on a large number of samples so a comparison was made on samples obtained from three different hospitals. The results are also shown in Table 8.

**Table 5. Comparision of results obtained on commercial quality control urine with manufacturer's stated values. Stated values in brackets.**

| Urine | Na mmol/l | K mmol/l | Creat mmol/l |
|---|---|---|---|
| Biotrol | 84<br>(84) | 42<br>(44) | 89<br>(90) |
| Q Pak | 107<br>(105) | 41<br>(40) | 34<br>(35) |
| Ortho | 90<br>(90) | 7.2<br>(6.9) | 9.7<br>(9.9) |

**Table 4. Comparision of results obtained on commercial quality control sera with manufacturer's stated values. Stated values are given in brackets.**

| Sera | Li mmol/l | Na mmol/l | K mmol/l | Cl mmol/l | $TCo_2$ mmol/l | Glu mmol/l | Urea mmol/l | Creat μmol/l | Anion Gap | Osm mosm/kg |
|---|---|---|---|---|---|---|---|---|---|---|
| Versatrol Alternate | | 153.6<br>(153) | 3.07<br>(3.1) | 113<br>(108) | 8.5 | 16.3<br>(16.7) | 21.1<br>(21.6) | 532<br>(539) | 35.2 | 315 |
| Monitrol II | 1.40<br>(1.48) | 129.4<br>(129) | 5.45<br>(5.5) | 115<br>(113) | 6.1 | 11.7<br>(11.5) | 12.7<br>(12.8) | 363<br>(358) | 13.7 | 280<br>(288) |
| Versatrol Normal | | 137.4<br>(137) | 4.82<br>(4.9) | 104<br>(102) | 5.9 | 4.1<br>(5.2) | 4.2<br>(4.3) | 238<br>(133) | 32.3 | 260 |
| Monitrol I E | 0.5<br>(0.53) | 137.2<br>(138) | 3.92<br>(3.95) | 103<br>(99) | 107 | 4.4<br>(4.9) | 4.7<br>(4.9) | 140<br>(132) | 27.4 | 264 |
| Q Pak II | | 154.8<br>(153) | 5.7<br>(5.7) | 121<br>(114) | 6.1 | 10.6<br>(11.4) | 16.0<br>(14.8) | 375<br>(422) | 33.4 | 314 |
| Versatrol Auto | | 145.9<br>(146) | 6.72<br>(6.8) | 120<br>(116) | 29.3<br>(35) | 15.2<br>(17.4) | 28.6<br>(26.4) | 548<br>(513) | 3.32 | 345 |
| Q Pak I | 1.53<br>(1.7) | 138.7<br>(139) | 4.4<br>(4.4) | 98<br>(95) | 7.5 | 3.7<br>(4.3) | 4.7<br>(4.4) | 111<br>(110) | 37.6 | 257 |
| Wellcome | 0.66<br>(0.7) | 142<br>(141) | 5.2<br>(4.9) | 101<br>(97) | 18.4<br>(18.4) | 5.9<br>(6.5) | 9.5<br>(9.9) | 177<br>(176) | 32.6 | 294 |
| Technicon I | | 150<br>(155) | 7.4<br>(7.8) | 124<br>(120) | 28<br>(30) | 11<br>(14) | 19<br>(20) | 560<br>(620) | | |
| " II | | 143<br>(143) | 4.6<br>(4.7) | 108<br>(105) | 24<br>(25) | 5.9<br>(6.7) | 9.5<br>(10) | 320<br>(340) | | |
| " III | | 123<br>(125) | 3.1<br>(3.0) | 81<br>(80) | 18<br>(15) | 3.3<br>(3.3) | 4.0<br>(4.1) | 105<br>(90) | | |

**Table 6. Comparison of results obtained on patient samples by Multistat I(Y) and Vickers M300(X) analyser and breakdown of data by Multistat I plate number. Each plate held 40 samples.**

| Day | Channel | Plate 1 | | | Plate 2 | | |
|---|---|---|---|---|---|---|---|
| | | r | Slope | Intercept | r | Slope | Intercept |
| 1 | Sodium | 0.99 | 0.98x | + 5.4 | 0.94 | 0.91x | + 16.6 |
| | Potassium | 0.94 | 0.88x | + 0.7 | 0.98 | 0.89x | + 0.6 |
| | Chloride | 0.96 | 0.97x | + 6.6 | 0.61 | 0.67x | + 37 |
| | Glucose | 0.97 | 0.99x | − 0.5 | 0.98 | 1.00x | − 0.6 |
| | Urea | 0.99 | 1.00x | + 0.1 | 0.99 | 1.03x | − 0.1 |
| 2 | Sodium | 0.98 | 0.96x | + 7.1 | 0.99 | 1.01x | + 1.1 |
| | Potassium | 0.99 | 0.94x | + 0.3 | 0.98 | 1.00x | + 0.1 |
| | Chloride | 0.93 | 1.01x | + 1.9 | 0.96 | 1.13x | − 6.2 |
| | Glucose | 0.99 | 1.03x | − 0.8 | 0.99 | 1.02x | − 4.7 |
| | Urea | 0.99 | 1.02x | − 0.1 | 0.99 | 1.04x | + 0.1 |
| 3 | Sodium | 0.98 | 0.99x | + 3.5 | 0.96 | 0.93x | + 9.6 |
| | Potassium | 0.98 | 0.99x | + 0.2 | 0.99 | 1.01x | + 0.1 |
| | Chloride | 0.93 | 1.09x | − 1.6 | 0.98 | 1.07x | − 2.9 |
| | Glucose | 0.99 | 1.00x | − 0.4 | 0.99 | 0.99x | − 0.4 |
| | Urea | 0.98 | 0.90x | + 1.9 | 0.99 | 1.05x | + 0.1 |

### Linearity

Each channel was tested for linearity over a range greater than that claimed by the manufacturer. Quality control sera reconstituted with aqueous standard and diluted to cover the range of each channel was used. Both sodium and potassium were linear with the line passing through the origin. The upper limit of each channel in the "Plasma" mode was 280 mmol/l for sodium and 28 mmol/l for potassium; these being the cut-out point of the electronic display. In the urine mode sodium was linear to over 450 mmol/l and potassium to the display cut-out point of 280 mmol/l.

Chloride is linear although the line passes through a point approximately 8 mmol/l from the origin. The upper limit of linearity is between 280 and 300 mmol/l.

Total $CO_2$ is linear over the range but does not pass through zero, having both an upper and lower limit of linearity. The range of apparent linearity is from 10-55 mmol/l although in fact there is a very slight curve over the whole slope. There is also a slope difference between aqueous and protein containing fluid; the slope with the latter being less steep.

Both the glucose and urea channels are linear with the line passing through the origin. The limitation to the linearity is determined by the enzyme concentration in the reagent and care must be taken if the reagent is "old" to make sure that the upper limit is adequate. Using the reagent as directed, glucose is linear up to 55 mmol/l and urea to 80 mmol/l.

The creatinine linearity is good and passes through the origin. In the plasma mode it is linear up to 1.5 mmol/l but since the display was changed to read μmol/l, the upper limit is restricted to 880 μmol/l. Any result over this will read 880 μmol/l and must be diluted and repeated.

Like the sodium and potassium channels, the lithium linearity is very good reaching the instrument cut off point at 28 mmol/l.

### Carryover

This was assessed using two quality control sera with high and low levels of analyte. Alternate sets of three high and three low sera were analysed and carryover for each channel calculated as in Broughton et al (2). A number of results were obtained for high - low and low - high sequences for each channel expressed as percentage interaction (I%). Because I% may be either positive or negative these results have been summarised in Table 9 by giving the root mean of the sum of the squares of the values in each sequence for each channel. Table 9 also gives the results for the chloride channel using degassed reagent which was seen to develop bubbles in the flow cell. Degassing was achieved by heating the reagent to 56°C before use. The reagent was then used at room temperature.

### Discussion

With the exception of the anion gap which was very imprecise, all the channels gave an acceptable precision suitable for clinical assessment of a patient. Precision between plates is frequently worse than one would expect from consideration of the within plate precision. This is probably due to standardisation and is most marked on the urea channel.

The comparisons made against commercial quality control sera for "accuracy" were on the whole satisfactory for most channels. The glucose channel gave slightly lower values than those quoted. This might be due to the fact that the glucose oxidase reacts with only one isomer of glucose and that although the samples were reconstituted at least two hours before use, they may not have equilibrated completely. The results were all high. The chloride content of the standards was checked independently and found to be correct. The total $CO_2$ results appear to lower at the high levels compared with the quoted values.

The comparisons with other analysers were on the whole satisfactory. The apparent lack of correlation between the SMA sodium and the Multistat I sodium is due to the narrow range of the normal serum sodium and the fact that unlike the Vickers M300 samples, the SMA Plus samples were not selected to cover a wide sodium range. There was a consistent

**Table 7. Comparison of results obtained on patient samples by Multistat I(Y) and Technicon SMA (X) analyser.**

| Day | n | Channel | r | Slope | Intercept |
|---|---|---|---|---|---|
| 4 | 15 | Sodium* | 0.91 | 1.07x | − 6.4 |
| | | Potasium | 0.97 | 1.20x | − 0.6 |
| | | Urea | 0.99 | 0.99x | − 0.2 |
| | | Creatinine | 0.98 | 0.91x | + 20.9 |
| 5 | 15 | Sodium* | 0.87 | 1.03x | − 1.5 |
| | | Potassium | 0.92 | 0.98x | + 0.3 |
| | | Urea | 0.99 | 0.98x | 0 |
| | | Creatinine | 0.99 | 0.93x | + 18.8 |
| 6 | 14 | Sodium* | 0.60 | 0.61x | + 57.0 |
| | | Potassium | 0.97 | 1.04x | − 0.2 |
| | | Urea | 0.99 | 0.99x | 0.1 |
| | | Creatinine | 0.93 | 0.90x | + 23.6 |
| 7 | 28 | Sodium* | 0.79 | 1.07x | − 6.0 |
| | | Potassium | 0.89 | 0.92x | + 0.4 |
| | | Urea | 0.99 | 1.09x | − 1.3 |
| | | Creatinine | 0.99 | 0.93x | + 14.4 |

**See comment on range of sodium results in Discussion section.*

bias in the glucose probably due to the need to transport the sample from one site to another but even then the results compared well. The chloride channel also showed a bias similar to that obtained with commercial quality control sera. It can be seen that there is a good correlation for total $CO_2$ over a range of 19-33 mmol/l. For osmolality, correlation is not good although many results are identical by both methods. It is possible that the correlation would have been better if the range of results had extended beyond the normal limits but the authors felt it appropriate to test the system with normal sera. It is also possible that a calculation allowing for the degree of dissociation of the constituent ions would yield a better value. The lithium results agree quite well although there is a slight bias for one of the hospitals.

Generally the instrument was linear above the claimed limits with total $CO_2$ the only problem. When the cut-off point was reached in the system the values were displayed at the cut off point. This was obviously a dangerous situation and the software has been subsequently modified to indicate that the value is "high".

The carryover on sodium and potassium was originally of concern because of the bore of the sample tube supplied. Changing this for a smaller bore tube improved the situation and the results shown here are acceptable. Carryover on the chloride channel reflects the inherent imprecision of the channel hence going from a low to high level the results are variable being sometimes positive and sometimes negative. It is not so bad when going from a high to low level. A possible explanation for this is that the total $CO_2$ is also high in the high samples and therefore going from a low to high level, the chloride channel has to cope with the possible inclusion of more bubbles in the system and when there is a decrease in level there is less "bubble" effect. The total $CO_2$ carryover is consistently high and no explanation can be offered since there is wide variation. It is possible that this could be due to the response time of the electrode or membrane but this is uncertain. Carryover on the glucose, urea and creatinine channels is low and frequently not detectable. This is because there is an automatic wash cycle in this unit and excess sample is completely aspirated before each channel takes its portion for analysis. The lithium carryover is worse than that of sodium and potassium and it should be remembered that a different pump is used. Urine carryover was assessed for sodium, potassium and creatinine and the performance was good, in relation to the large differences in values. Creatinine however suffered from a "rebound" effect with a considerable number of results being negative.

During the evaluation various parts of the system developed faults most of which were obvious and were remedied rapidly by the company. The faults were: failure of pump on chloride/ total $CO_2$ unit requiring new bearings, failed gas pressure switch on flame unit, fault in the sample light sensor block requiring re-adjustment and a faulty display unit on the chloride/total $CO_2$ unit. Over one period of four days the computer unit caused considerable problems and finally had to be replaced. The problems began with interupts during running and culminated in a number of occasions when the program lost the quality control data completely. While processing the data for analysis it was discovered that during the period immediately prior to the computer replacement and subsequently that the plate backup and the plate printout have shown discrepancies in a single line of analytical results on at least eight occasions.

**Table 8. Comparison of results obtained on patient samples by Multistat I(Y) and other methods (X) as given.**

| Channel | n | r | Slope | Intercept | Compared Method |
|---|---|---|---|---|---|
| Total $CO_2$ | 58 | 0.91 | 0.91x | + 2.2 | Technicon AAI |
| Osmolality | 30 | 0.64 | 0.67x | + 90 | Wescor Vapour phase |
| " | 22 | 0.83 | 0.88x | + 32 | osmometer |
| Lithium | 10 | 0.95 | 1.09x | – 0.1 | IL 343 |
| " | 13 | 0.97 | 0.84 | + 0.2 | Corning 450 |
| " | 17 | 0.97 | 0.79 | + 0.1 | EEL 120 |

There were inherent problems in the software package during the evaluation period which interfered with the data collection phase. The manufacturers have subsequently modified the software.

Although patient samples were not run routinely during this trial, in our experience a throughput of 200 samples per day could be achieved comfortably.

**Safety aspects**

In order to prevent spillage of material onto the printed circuit board of the teletype keyboard a hermetically sealed unit was installed. (This is an expensive modification since this keyboard cost approximately £1,500). This minimises the electrical hazard but not the problem of sterilisation. The machine was designed to be used by a single operator filling the vials and entering the data via the keyboard. Contamination of the keyboard was inevitable and sterilisation is not easy. It was suggested that a team of two people would be more suitable at the filling and data entering stage. The urea, creatinine and glucose unit was standardised by aqueous material contained in sealed glass ampoules. These should be discontinued and replaced by plastic containers to avoid the hazard produced by the cracked glass.

**Instruction manuals**

At the training course one set of instruction manuals for each of the analytical units was provided. These contained some errors and the instrument company is replacing it in due course. A software training book for the system provided during the second stage of the training was very helpful. A complete instruction book for the whole system was not provided because again this was in the process of being written.

For most purposes the information provided has been adequate to run the system and deal with minor faults. Errors discovered in the existing instruction manuals show that major problems require a complete instruction manual. This is being provided by the instrument company.

### Conclusions

The overall analytical performance of the system was satisfactory bearing in mind the need to degas the chloride reagent. The authors feel that the calculated parameters may be useful in terms of quality control within the laboratory but are of little practical use to the clinician.

The satisfactory performance together with the software control and facilities such as cumulative reporting make the system attractive to the medium sized hospital laboratory.

**Table 9. Carryover of each channel**

| Channel | No. of observations | Root mean of the sum of squares of percentage interaction (1%) | |
|---|---|---|---|
| Serum | | Low – High | High – Low |
| Sodium | 11 | 0.83 | 1.48 |
| Potassium | 11 | 0.88 | 0.53 |
| Chloride | 11 | 6.10 | 1.72 |
| Chloride (reagent degassed) | 11 | 1.56 | 0.92 |
| Total $CO_2$ | 11 | 18.10 | 15.10 |
| Glucose | 11 | 0.86 | 1.51 |
| Urea | 11 | 1.20 | 0.72 |
| Urine | | | |
| | (High – low 6) | | |
| Sodium | 5 | 0.84 | 0.46 |
| Potassium | 5 " | 0.32 | 0.35 |
| Creatinine | 5 " | 3.18 | 2.80 |

ACKNOWLEDGEMENTS
The authors thank the Department of Health and Social Security for providing the system to be tested, Dr Burnett of St Albans City Hospital and Dr Cheng of Watford General Hospital for their help with the comparative studies and the staff of Instrumentation Laboratory Ltd for their co-operation. The advice of Dr S S Brown of the Clinical Research Centre, Harrow and Dr I W Percy-Robb of Edinburgh Royal Infirmary is also gratefully acknowledged.

REFERENCES
[1] Gibson P F, Calzi C, Musetti A, and Caliri R. X International Congress of Clinical Chemistry, Mexico City, 1978.
[2] Broughton P M G, Gowenlock A H, McCormack J J, and Neill D W (1974) *Annals of Clinical Biochemistry*, **11**, 207-218.

### Multilevel analysis of variance used to partition components of optical imprecision in an analyser system with disposable cuvettes*

**Douglas M. Fast, Eric J. Sampson, Carl A. Burtis****
*Clinical Chemistry Division, Bureau of Laboratories, Center for Disease Control, Public Health Service, U.S. Department of Health and Human Services, Atlanta, Georgia 30333, USA.*

### Introduction

With the increasing use of disposable cuvettes in modern spectrophotometric instrumentation it is vital for the analyst to be aware of the various types of errors that can be introduced into the analytical process. Other investigators have described these errors and their propagation in spectrometric systems [1-5] or have examined random errors in various specific components of their systems [6-10]. On the basis of these studies, various professional organisations have proposed guidelines for spectrometric instruments [11-14]. However, for an analytical system using a disposable rotor containing a large number of cuvettes which is used only once and discarded, a statistical technique must be implemented to quantitate random optical error, to check actual instrument performance against manufacturer's specifications, to assess the quality of incoming supplies for the centrifugal analyser, and to provide criteria for explicit operational practices in the use of the analyser.

Analysis of variance (ANOVA) has previously been used in assigning magnitudes of error to sources within a multiunit instrument or method [15], but has been usually limited to an examination of only two or three variables. This approach has been extended to a three-level nested ANOVA which separates the random optical noise component from errors in absorbance associated with possible changes in the physical parameters of the disposable cuvettes. These errors include variations in the absorbance within-cuvettes, between-cuvettes, between rotors, and between manufacturer's lots of rotors.

### Materials and methods

### *Instrument*

The centrifugal analyser system evaluated was the Multistat III micro centrifugal analyser [3] (Instrumentation Laboratory, Lexington, MA 02173 USA). The analyser system consists of two modules, an automated loader and the spectrophotometric analyser. The analyser uses disposable plastic cuvettes (20 per rotor) for incubation, mixing, and measurement of absorbance (Figure 1). The 0.5 cm optical cell is formed by moulding clear windows in the cuvette top and bottom surfaces. Of the 20 cuvettes in the rotor, the first is used as the reference cuvette, and the remaining 19 are used for any combination of standards and samples necessary. Narrow bandpass interference filters are used in the photometer to isolate the spectral range of interest. Transmitted radiation is measured by using a photomultiplier tube and an

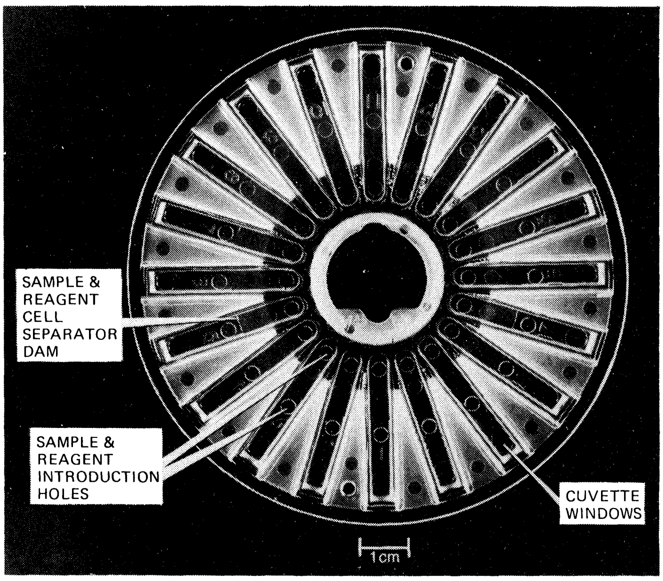

*Figure 1. Disposable rotor used with Multistat III analyser system (top view). Cuvette windows are an integral part of the top and bottom walls of each cuvette. Upon acceleration, sample flows over the separator dam and mixes with reagent to initiate chemical reaction.*

*Presented in part at the Pittsburgh Conference on Analytical Chemistry and Applied Spectroscopy, Cleveland, Ohio, 1979.*
***Present address: Chemical Technology Division, 4500N, Oak Ridge National Laboratories, Oak Ridge, TN 37830.*

